# Electromagnetic field treatment increases purinergic receptor P2X7 expression and activates its downstream Akt/GSK3β/β-catenin axis in mesenchymal stem cells under osteogenic induction

**DOI:** 10.1186/s13287-019-1497-1

**Published:** 2019-12-21

**Authors:** Yingchi Zhang, Wenkai Li, Chaoxu Liu, Jiyuan Yan, Xuefeng Yuan, Wei Wang, Huaixi Wang, Hua Wu, Yong Yang

**Affiliations:** 1 Department of Orthopedics, Tongji Hospital, Tongji Medical College, Huazhong University of Science and Technology, 1095 Jiefang Avenue, Wuhan, 430030 China; 2 Department of Traumatic Surgery, Tongji Hospital, Tongji Medical College, Huazhong University of Science and Technology, 1095 Jiefang Avenue, Wuhan, 430030 China; 3 Department of Spine and Spinal Cord Surgery, Zhengzhou University People’s Hospital, Henan Provincial People’s Hospital, 7 Weiwu Road, Zhengzhou, 450003 China

**Keywords:** Electromagnetic fields (EMFs), Purinergic receptor P2X7, Human bone marrow mesenchymal stem cells (h-MSCs), Osteogenic differentiation, Akt/GSK3β/β-catenin signaling pathway

## Abstract

**Background:**

Imbalance in bone formation and resorption is a crucial component of the pathological process leading to osteoporosis. Electromagnetic fields (EMFs) have been reported to be beneficial to osteogenesis, although the exact mechanism has not been fully clarified. Purinergic receptor P2X7 is expressed in osteoblasts and is reported to participate in the regulation of bone metabolism.

**Objectives:**

To elucidate the link between EMFs and P2X7 expression and investigate its potential as a novel therapeutic target in osteoporosis.

**Method:**

We investigated the effect of EMFs on P2X7 expression and downstream signaling in human bone marrow mesenchymal stem cells (h-MSCs). We also established an ovariectomized (OVX) osteoporosis rat model to evaluate the therapeutic efficacy of combining EMFs with P2X7 agonists.

**Results:**

EMF treatment increased P2X7 expression in h-MSCs under conditions of osteogenic induction but not under regular culture conditions. P2X7 or PI3K/Akt inhibition partially inhibited the pro-osteogenic effect of EMF and lowered the EMF-stimulated activity of the Akt/GSK3β/β-catenin axis. No additive effect of this suppression was observed following simultaneous inhibition of P2X7 and PI3K/Akt. EMF treatment in the presence of a P2X7 agonist had a greater effect in increasing osteogenic marker expression than that of EMF treatment alone. In the OVX osteoporosis model, the therapeutic efficacy of combining EMFs with P2X7 agonists was superior to that of EMF treatment alone.

**Conclusions:**

EMF treatment increases P2X7 expression by h-MSCs during osteogenic differentiation, leading to activation of the Akt/GSK3β/β-catenin axis, which promotes the osteogenesis. Our findings also indicate that combined EMF and P2X7 agonist treatment may be an effective novel strategy for osteoporosis therapy.

## Introduction

Osteoporosis, which literally means porous bone, is a disease in which the density and quality of bone are reduced. Currently, there are millions of osteoporosis patients worldwide, most of which are postmenopausal women. It is characterized by reduced bone mass and micro-architectural degradation of bone tissue, resulting in increased bone fragility and higher fracture risk [1]. Common treatments for osteoporosis include dietary and lifestyle changes as well as pharmacologic therapies, such as teriparatide, denosumab, and bisphosphonates [2, 3]; however, these interventions are limited by multiple side-effects, high cost, and low patient compliance. Although the specific pathogenesis of osteoporosis is unclear, there is increasing evidence that dysplasia of bone marrow stromal cells (BMSCs) is major cause of structural abnormalities in osteoporosis bones [4–6]. BMSCs are self-renewable, multipotent stem cells that can be differentiated into different lineages of chondrocytes, osteoblasts, adipocytes, and other mesenchymal tissues, after culturing with appropriate hormonal inducers or growth factors under appropriate conditions [7, 8]. Uncoupling between osteoblast and osteoclast activity and/or loss of the balance between osteogenic differentiation and adipogenic differentiation of BMSCs lead to osteoporosis.

As a clinically safe, effective, and noninvasive treatment, electromagnetic field (EMF) therapies have been well received during recent decades. In the field of orthopedics, EMF therapy is commonly used to treat bone fractures [9] and musculoskeletal disorders, including osteoarthritis and rheumatoid arthritis [10]. Based on pre-clinical studies and prospective clinical trials, the Food and Drug Administration, USA, approved pulsed EMF therapy as a safe and effective method for treating delayed union or nonunion fractures [11, 12]. In the last few years, EMFs have been widely reported to positively affect the balance of osteoblast and adipocyte differentiation of mesenchymal stem cells [13–15] and the balance between bone formation and bone resorption [16], which are critical components of the development of osteoporosis. These reports indicated that EMFs can be used to improve the state of osteoporosis. Previous studies confirmed that EMF treatment directly induced [17] or accelerated [18] the osteogenic differentiation of BMSCs. However, the mechanism by which EMFs induce or accelerate osteogenic differentiation of MSC remains to be fully elucidated.

Extracellular nucleotides, such as ATP and UTP, as soluble factors released into cellular matrix in response to mechanical stimuli, signal in the autocrine or paracrine manner through specifically binding cell surface P2 receptors [19–21] There is increasing evidence that extracellular nucleotides play an important role in bone metabolism [21–23]. Depending on molecular structure and activation of signal pathway, P2 purinergic receptors fall into two categories in mammalian cells, including seven P2X subtypes and eight P2Y subtypes [24].

In particular, the purinergic receptor P2X7, a ligand-gated ion channel, is closely involved in bone remodeling and mechanical transduction. P2X7 knockout resulted in an osteopenic phenotype, in which the formation of periosteal bone is reduced in long bones while there is no significant difference in length of bone or trabecular bone resorption [25]. Further in vivo loading experiments showed that appositional growth of the long bone is reduced and skeletal responses to mechanical loading are weakened in these mice [26]. An in vitro study has since indicated that shockwaves enhance osteogenic differentiation of human mesenchymal stem cells through ATP release and P2X7 activation [27]. P2X7-induced membrane blebbing promotes osteogenesis and mineralization of postmenopausal bone marrow-derived mesenchymal stem cells [28]. However, a link between EMF exposure and P2X7 expression in osteogenic differentiation of MSCs has not yet been reported.

In this study, we found that P2X7 expression was increased after EMF exposure. Furthermore, the increased P2X7 expression contributed to MSC osteogenic differentiation via the Akt/GSK3β/β-catenin axis. We also found that utilization of P2X7 agonists improved the therapeutic effects of EMF in osteoporosis.

## Materials and methods

### EMF device

The EMF generation device which comprises a waveform generator, amplifier, oscilloscope, and Helmholtz coils was designed and manufactured by the Naval University of Engineering of China (Wuhan, China). Signals produced by the waveform generator were amplified and then output to the Helmholtz coils. The Helmholtz coils (diameter 30 cm, 15 cm apart) which were wound with coated copper wire (diameter 0.8 mm) generated the EMF which is used in the following experiment. For the in vitro experiments, the Helmholtz coils were vertically placed in a CO_2_ incubator (Thermo Scientific, Wilmington, DE, USA) (Additional file 1: Figure S1A). For the in vivo experiments, the Helmholtz coils were vertically placed in a ventilated environment at 26 °C and the Plexiglas cages (length 35 cm, width 30 cm, height 45 cm) were placed between the coils, in which rats were housed individually with free access to clean tap water and standard rodent chow (Animal Center of Tongji Medical College, Wuhan, China) (Additional file 1: Figure S1B). EMF generated by the device were at frequency range of 0–100 Hz and an intensity range of 0–5.0 mT. A sinusoidal EMF was used as it showed a satisfactory effect in our previous studies [29, 30]. The intensity of the EMF was measured using a gauss meter (GM55A; TinDun Industry, Shanghai, China). The uniformity of the EMF was approximately 90% in the 7 cm of the spherical region (from the coil center to the origin).

### Human MSC culture and stimulation

Human bone marrow MSCs (h-MSC) (Cell Bank of the Chinese Academy of Sciences, Shanghai, China) were identified by detecting mesenchymal stem cell surface markers through flow cytometry (Additional file 1: Figure S2A) and evaluating the potential for differentiation towards adipocytes, osteoblasts, and chondrocytes (Additional file 1: Figure S2A). The h-MSCs were cultured in Gibco Dulbecco’s modified Eagle medium/Ham’s F-12 (DMEM/F12) supplemented with 10% FBS and 100 U/mL penicillin–streptomycin (Gibco, USA) under condition of 5% CO_2_, 37 °C, and 100% humidity. Upon the cells were approximately 90% confluence, 0.25% trypsin (Gibco, USA) were used to detach the cells, which then passaged at a ratio of 1:2 or 1:3. Passages between 3 and 5 were used in the following experiment. For the EMF treatment, MSCs were exposed for 8 h per day. To inhibit P2X7 or PI3K/Akt activity, cells were treated with 5 μM P2X7 blocker A740003 (Sigma–Aldrich, St. Louis, MO, USA) or 10 μM PI3K/Akt inhibitor LY294002 (Sigma–Aldrich).

### RNA extraction and qRT-PCR analysis

Total RNA was extracted from human mesenchymal stem cells (h-MSCs) using TRIzol reagent (Invitrogen, Carlsbad, CA, USA). The concentration and purity of RNA samples were assessed by spectrophotometric analysis. Three micrograms of RNA was used to reverse transcription by using an EasyScript First-Strand cDNA Synthesis Super Mix kit (TransGen Biotech, Beijing, China). The relative mRNA expression of human P2X7, RUNX2, ALP, OPN, and GAPDH (Invitrogen) was evaluated by quantitative real-time PCR (qRT-PCR) by using the BioRad myiQ2 Sequence Detection System (BioRad, Hercules, CA, USA) and TransStart Eco Green qPCR Super Mix (TransGen Biotech, Beijing, China). Primers were purchased from Invitrogen, and the sequences were listed in Additional file 1: Table S1. Cycle conditions followed primers’ introduction. Relative gene expressions were normalized with GAPDH and analyzed by the 2^−ΔΔCt^ method.

### Western blot analysis

The h-MSCs were cultured in 6-well plates (5 × 10^5^ cells/well) in expansion medium for 24 h. After incubation and stimulation, cells were washed using ice-cold PBS (Boster Biol Tech, Wuhan, China) and centrifuged at 1000 rpm for 10 min. Total proteins were isolated by lysing cells in radioimmunoprecipitation assay lysis buffer containing 1% phosphatase inhibitors (Boster Biol Tech) for 30 min on ice. Lysates were centrifuged at 13,000 rpm for 15 min at 4 °C, then the harvested supernatants were stored at − 20 °C prior to analysis. Nuclear and Cytoplasmic Protein Extraction Kit (Beyotime, Shanghai, China) was used to extract cytoplasmic and nuclear proteins according to the manufacturer’s protocol. BCA protein assay kit (Applygen, Beijing, China) was used to assess protein concentration. Proteins in equivalent amount from each sample were separated by 10% SDS-PAGE (BioRad) electrophoresis and then transferred onto polyvinylidene fluoride (PVDF) membranes (Merck Millipore, Billerica, MA, USA). 5% BSA (Boster Biol Tech) in 1× TBST (0.1% Tween-20) was used to block the membranes for 1 h at room temperature. After blocking, membranes were incubated with primary antibodies (Cell Signaling Technologies Inc., Milan, Italy) against P2X7, RUNX2, OPN, Akt, p-Akt, GSK3β, p-GSK3β, E-cadherin, Fibronectin, Vimentin, Snail, ALDH1A1, β-catenin, lamin B1 (all 1:1000), and GAPDH (1:400) at 4 °C overnight. The next day, membranes were washed three times with TBST on ice for 10 min and then incubated with HRP-conjugated goat anti-mouse or goat anti-rabbit IgG secondary detection antibodies (Boster Biol Tech) (1:5000) at room temperature for 1 h. Membranes were washed three times in TBST for 10 min each. Enhanced chemiluminescence (ECL) reagent was used to detect immunostained protein bands through autoradiography. Image-Lab software (BioRad, Hercules, CA, USA) was used to analyze the band intensities. Relative expression was normalized using GAPDH or lamin B1 as a loading control, and data were presented as a percentage of the expression of the reference genes.

### Alizarin red S staining and Oil red O staining

To induce osteogenic differentiation, h-MSCs were seeded on plastic dishes (diameter 35 mm) with a density of 2 × 10^4^ cells/cm^2^ and cultured for 7 to 21 days in human osteogenic induction mediums (Cyagen). The formulation of the human osteogenic induction medium is 175 mL h-MSC osteogenic differentiation basal medium with 20 mL fetal bovine serum, 2 mL penicillin–streptomycin, 2 mL glutamine, 400 μL ascorbate, 2 mL β-glycerophosphate, and 20 μL dexamethasone.

For Alizarin red S staining, h-MSCs were washed with PBS and fixed with 4% paraformaldehyde at room temperature for 30 min. Then the fixed cells were washed with PBS and stained with 1 mL Alizarin red S working solution for 3–5 min. After two to three times rinse with PBS, then the cells were photographed.

### Ovariectomy models

Sprague Dawley rats were purchased from the Experimental Animal Center of Huazhong University of Science and Technology (Wuhan, China) Twenty-four female Sprague Dawley rats were randomly divided into four groups: sham-operated controls, OVX controls, OVX with EMF stimulation, and OVX with both EMF stimulation and BzATP administration. Rats underwent the sham operation or ovariectomy at age of 8 weeks (weighing 200–220 g) and recovered for 2 weeks. All experimental procedures were performed on the animals with the official approval of the Ethics and Animal Research Committee of Huazhong University of Science and Technology. From 10 weeks of age, the sham-operated controls and OVX controls received daily intraperitoneal injections of 0.9% NaCl. The OVX+EMF and OVX+EMF+BzATP groups were exposed to EMFs (15 Hz/1 mT) for 8 h per day (from 9:00 AM to 5:00 PM). The OVX+EMF+BzATP group administrated daily of BzATP (5 mg/kg/day) by intraperitoneal injection. All rats were euthanized after 12-week treatment. The right femur and tibia of these rats were excised and fixed with 4% paraformaldehyde for further analysis.

### Micro-CT scanning and analysis

Scanco viva CT 40 instrument (Scanco, Brüttisellen, Switzerland) was used to scan the right femur and tibia excised from all groups of rats. The micro-structure of above bones was analyzed as reported in our previous study [30]. Briefly, contiguous cross-sectional images (10.5 μm) were obtained at 70 kV and 113 mA. The constant threshold for trabecular and cortical bone was set at 180 and 220, respectively, in order to distinguish the bone from the bone marrow. For trabecular bones, the region of interest (ROI) began at 10 slices (105 μm) below the lowest point of the growth plate and extended downward for 200 slices (2100 μm). For the cortical bones, the ROI was selected from the whole femoral. The trabecular morphometry parameters were analyzed including the relative bone volume (bone volume/total volume, BV/TV).

### Statistical analysis

All data were presented as mean values ± standard deviation (SD). Differences among each time points or among each treatment groups were determined by one-way analysis of variance (ANOVA). Following the one-way ANOVA, significances between every pair of time points or treatment groups were determined by Tukey’s post hoc analysis. Statistical analysis was performed using the software of Statistical Package for Social Sciences (SPSS 15.0 for Windows; SPSS, Chicago, IL). *P* < 0.05 was accepted as significant difference.

## Results

### Various frequencies of EMFs increased P2X7 expression in h-MSCs under osteogenic differentiation

**Fig. 1:**
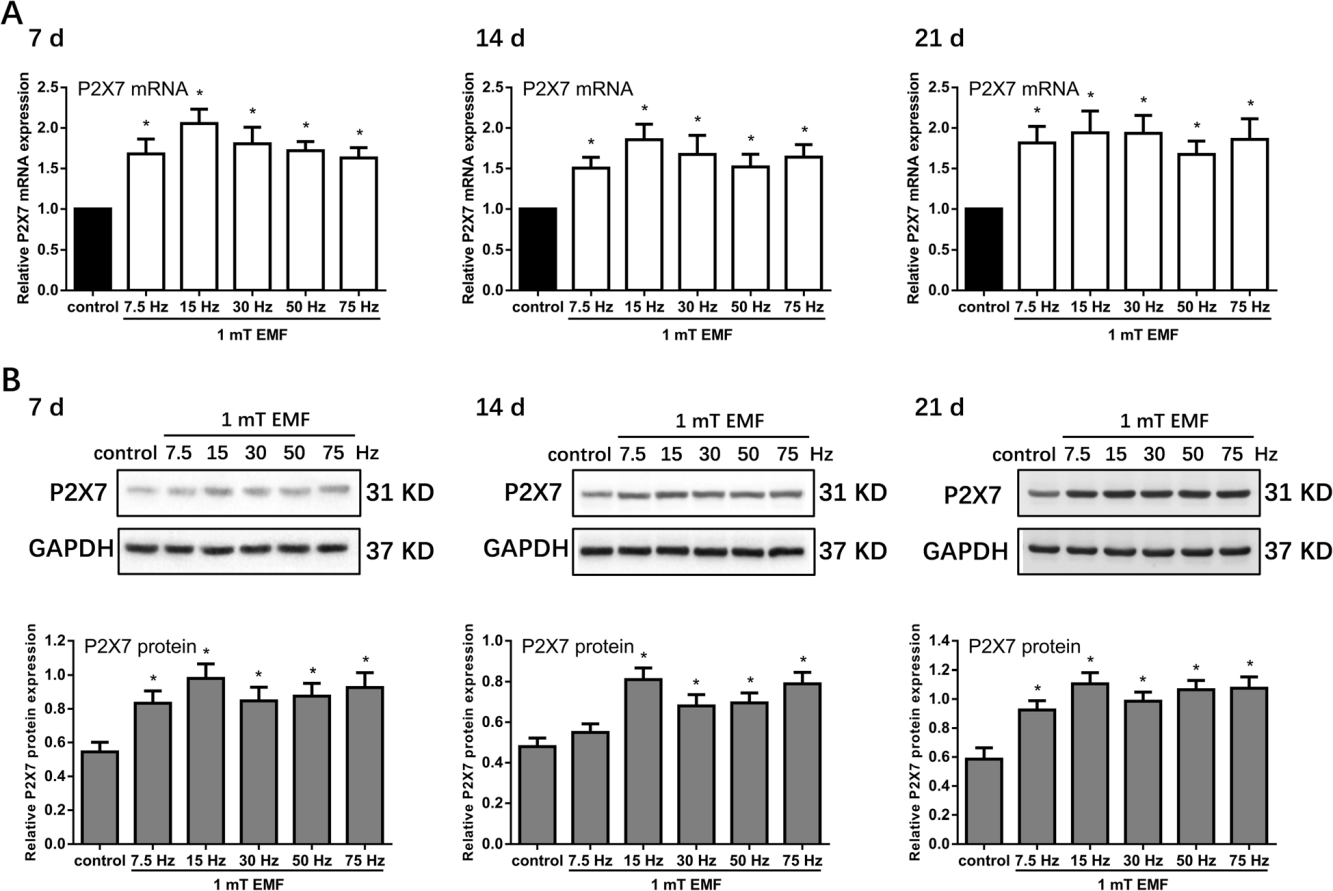
Effects of exposure to various frequencies of EMF on P2X7 expression in osteogenic differentiated h-MSCs. **a** qRT-PCR and **b** Western blot analysis of P2X7 expression in h-MSCs exposed to various frequencies of EMF (7.5, 15, 30, 50, and 75 Hz of 1 mT) for 7, 14, and 21 days. The mRNA and protein expression of P2X7 was normalized against GAPDH. Data represent means ± SD. Statistically significant differences are indicated; *n* = 3; *P < 0.05, vs. control

h-MSCs were exposed to various frequencies of EMFs (7.5, 15, 30, 50, and 75 Hz/1 mT) in the presence of osteogenic induction medium for 7, 14, and 21 days. P2X7 expression was detected at the mRNA and protein levels by qRT-PCR and Western blot analysis, respectively. We found that 1 mT EMFs at all these frequencies (7.5, 15, 30, 50, and 75 Hz) increased P2X7 expression to varying degrees at both the mRNA and protein levels from 7 to 21 days. Among the frequencies, 15 Hz exhibited the optimal ability to increase P2X7 expression (Fig. 1a, b) and was therefore selected for use in further research.

### The EMF-induced P2X7 expression in h-MSCs was detected during osteogenic differentiation but not in regular culture

**Fig. 2:**
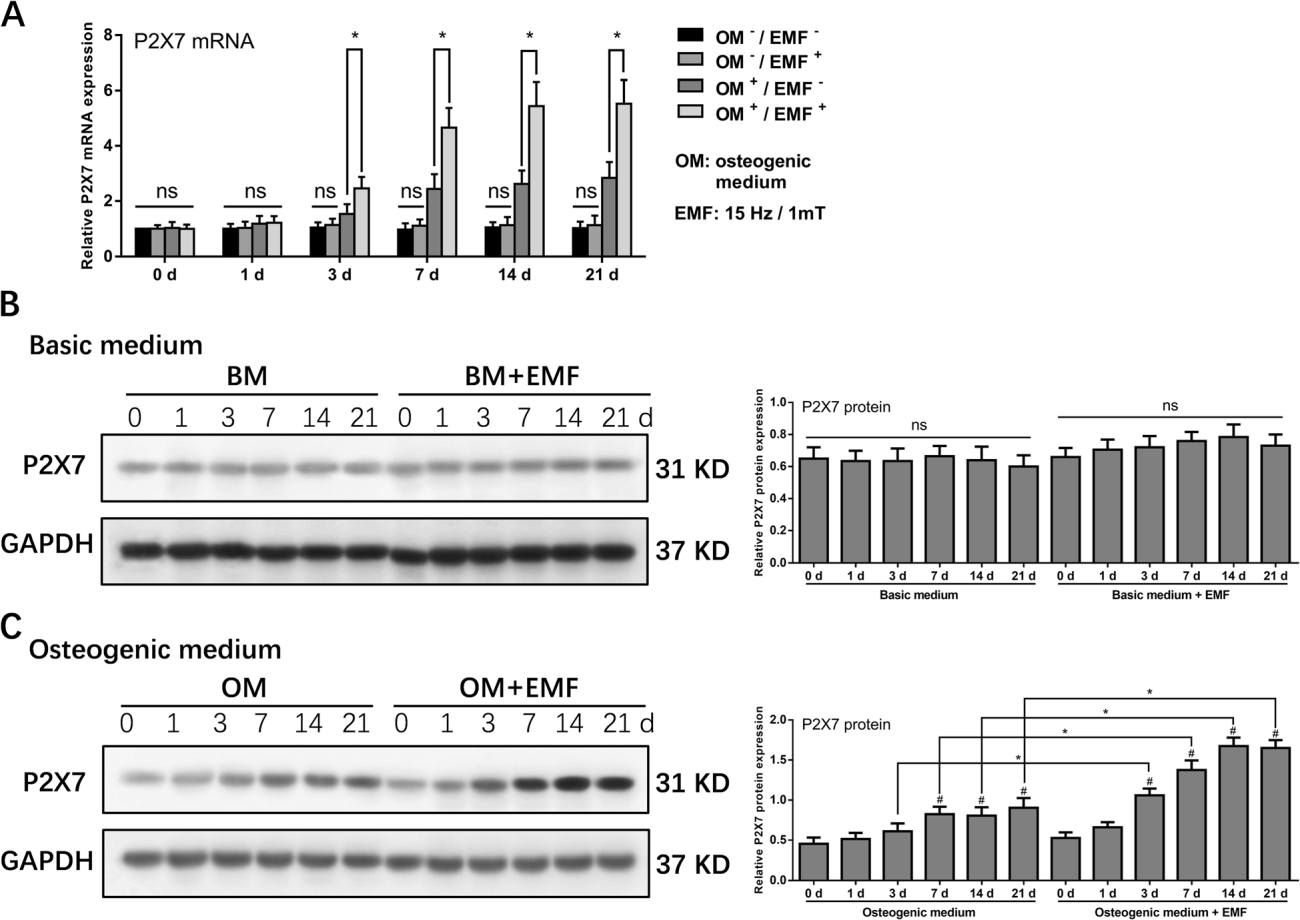
P2X7 expression in h-MSCs under regular culture and osteogenic differentiation. **a** qRT-PCR analysis of P2X7 expression in h-MSCs cultured in basic medium, basic medium+15 Hz/1 mT EMF, osteogenic medium, and osteogenic medium+15 Hz/1 mT EMF for 0, 1, 3, 7, 14, and 21 days. P2X7 mRNA expression was normalized against GAPDH. Western blot analysis of P2X7 expression in h-MSCs treated with **b** basic medium, basic medium+15 Hz/1 mT EMF, **c** osteogenic medium and osteogenic medium+15 Hz/1 mT EMF for 0, 1, 3, 7, 14, and 21 days. P2X7 protein expression was normalized against GAPDH. Data represent means ± SD. Statistically significant differences are indicated; *n* = 3; *P < 0.05, vs. control

To examine the conditions and timing of EMF-stimulated P2X7 expression, we cultured h-MSCs with basic medium or osteogenic medium for 0, 1, 3, 7, 14, and 21 days prior to analysis of P2X7 expression. The qRT-PCR analysis showed that EMF did not affect h-MSC expression of P2X7 mRNA under basic medium culture conditions. In contrast, under osteogenic culture conditions, h-MSC expression of P2X7 mRNA was slightly increased (approximately 20%) on day 3 and was increased by more than twofold on days 7 to 21. This effect was significantly enhanced by exposure to EMFs. h-MSC cultured in osteogenic medium combined with EMF exposure exhibited a marked increase (80%) in P2X7 mRNA expression on day 3, with further increases (four- to fivefold) in P2X7 expression detected on days 7 to 21 (Fig. 2a). In h-MSCs cultured in basic medium, Western blot analysis showed no significant differences in P2X7 protein expression at the various time points, with or without EMF exposure (Fig. 2b). In h-MSCs cultured in osteogenic medium without EMF exposure, P2X7 protein expression increased by 30% on days 7 to 21, but was increased by 100 to 200% on days 3 to 21 in the presence of EMFs (Fig. 2c). These results suggested that EMFs significantly promote P2X7 expression in h-MSCs during osteogenic differentiation.

### P2X7 expression was linked to EMF-enhanced osteogenic differentiation in h-MSCs

**Fig. 3:**
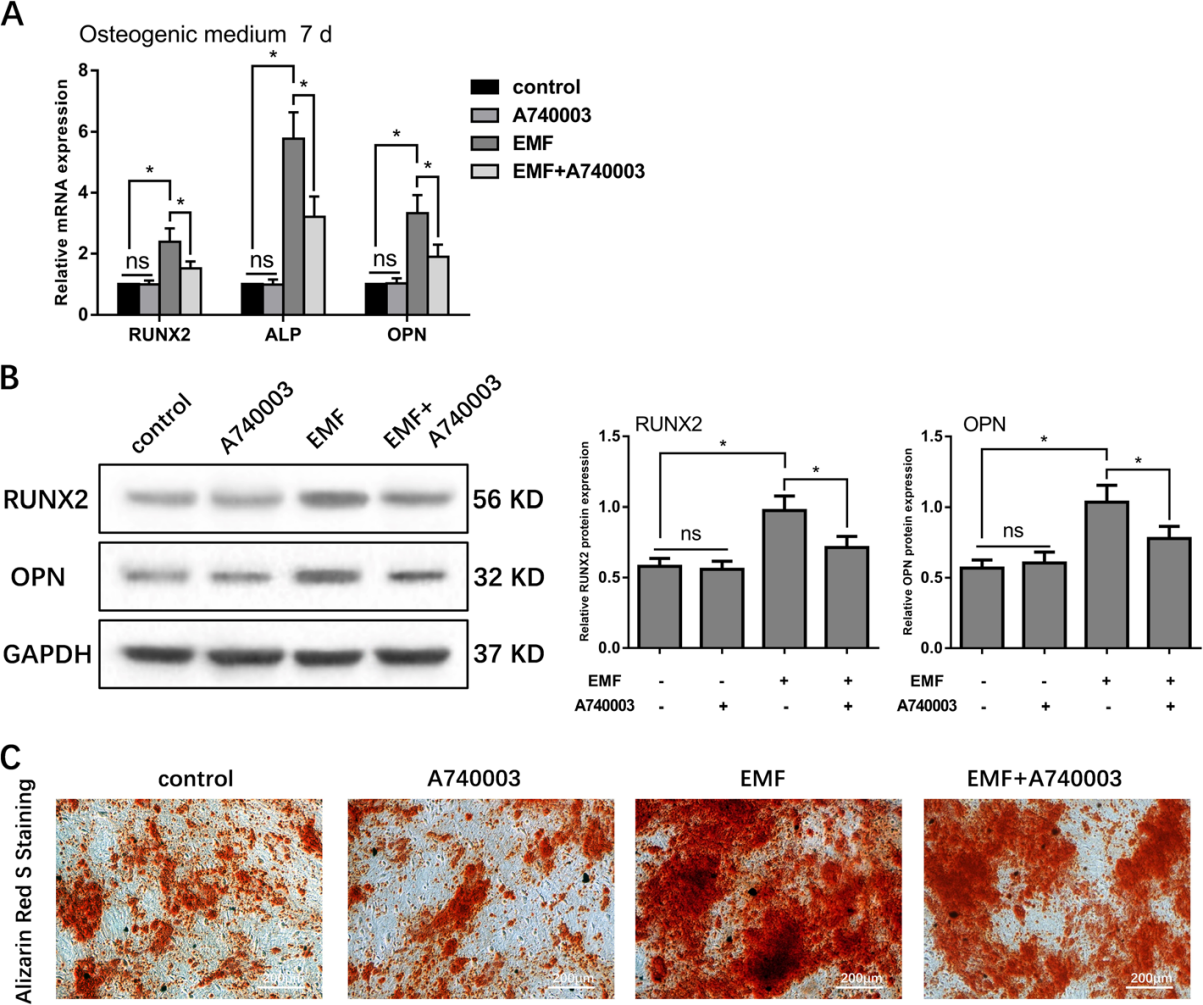
Role of P2X7 in EMF-enhanced osteogenic differentiation in h-MSCs. **a** qRT-PCR analysis of osteogenic markers (RUNX2, ALP, and OPN) expression in h-MSCs cultured in osteogenic medium, osteogenic medium+5 μM A740003, osteogenic medium+15 Hz/1 mT EMF, and osteogenic medium+15 Hz/1 mT EMFs+5 μM A740003 for 7 days. The mRNA expression of RUNX2, ALP, and OPN was normalized against GAPDH. **b** Western blot analysis of osteogenic marker (RUNX2 and OPN) expression in h-MSCs cultured in osteogenic medium, osteogenic medium+5 μM A740003, osteogenic medium+15 Hz/1 mT EMF, and osteogenic medium+15 Hz/1 mT EMFs + 5 μM A740003, respectively, for 7 days. The protein expression of RUNX2 and OPN normalized against GAPDH. **c** Alizarin red S staining of h-MSCs treated with osteogenic medium, osteogenic medium + 5 μM A740003, osteogenic medium + 15 Hz/1 mT EMFs, and osteogenic medium + 15 Hz/1 mT EMFs + 5 μM A740003 for 7 days. Data represent means ± SD. Statistically significant differences are indicated; *n* = 3; *P < 0.05, vs. control

Our previous study showed that 15 Hz/1 mT EMF exposure significantly promoted osteogenic differentiation of rat MSCs [31]. In this study, we verified the pro-osteogenic effect of EMF exposure on h-MSCs cultured in osteogenic medium. Furthermore, we used A740003, a specific P2X7 antagonist, to investigate whether this effect was related to P2X7 expression. The qRT-PCR analysis showed that EMF exposure significantly increased (1.5- to 4-fold) the expression of the osteogenic-related genes, RUNX2, ALP, and OPN, in h-MSCs cultured in osteogenic medium for 7 days. This effect of EMF exposure was significantly lowered (by 40%) by treatment with 5 μM A740003, although the basal expression of RUX2, ALP, and OPN was not reduced (Fig. 3a). The results of Western blot analysis were consistent with the qRT-PCR results. The RUNX2 and OPN protein expression was doubled by EMF treatment for 7 days, but increased by only 20 to 30% by EMF exposure in the presence of A740003. There were no significant differences in the expression of osteogenic markers between the A740003 only and control groups (Fig. 3b). After osteogenic culture for 7 days, Alizarin red S staining showed that h-MSCs in the EMF group generated much more calcium nodules than those in the control group. This effect of EMFs was weakened in the presence of 5 μM A740003. Treatment with A740003 alone had no influence on the number of Alizarin red S-stained nodules compared with the control group (Fig. 3c). These results suggested that EMFs promote the osteogenic differentiation of h-MSCs via a mechanism that is partially related to the expression of P2X7.

### The EMF-induced upregulation of P2X7 expression promoted osteogenic differentiation of h-MSCs via the Akt/GSK-3β/β-catenin signaling pathway

**Fig. 4:**
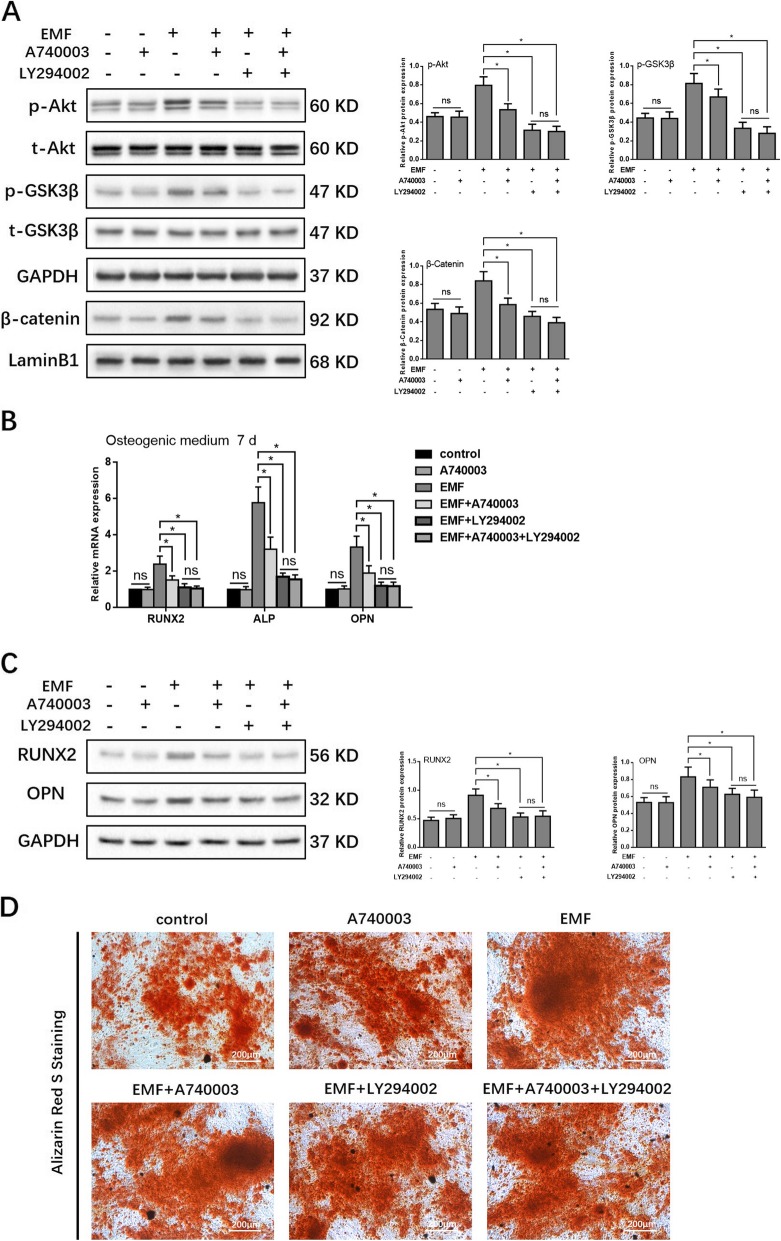
Activation of the Akt/GSK-3β/β-catenin signaling pathway in osteogenic differentiated h-MSCs by upregulated P2X7 expression induced by EMF exposure. **a** Western blot analysis of the effect of EMF on protein expression of p-Akt (Ser473), total Akt, p-GSK3β (Ser9), total GSK3β, and nuclear β-catenin in the presence of A740003 or/and LY294002. **b** qRT-PCR analysis of the effect of EMF exposure in the presence of A740003 or/and LY294002 on mRNA expression of RUNX2, ALP, and OPN. **c** Western blot analysis of the effect of EMF in the presence of A740003 or/and LY294002 on protein expression of RUNX2 and OPN. **d** Alizarin red S staining of osteogenic induced h-MSCs exposed to EMF in the presence of A740003 or/and LY294002 for 7 days. Data represent means ± SD. Statistically significant differences are indicated; *n* = 3; *P < 0.05, vs. control

We next investigated the role of the Akt/GSK-3β/β-catenin pro-osteogenic signaling axis in the mechanisms by which EMF-induced upregulation of P2X7 expression promotes osteogenic differentiation. Stimulation of h-MSCs with EMF resulted in increased (approximately 40%) levels of phosphorylated Akt, phosphorylated GSK-3β, and nuclear β-catenin, while this effect was reduced when the P2X7 was blocked by A740003. The EMF-induced upregulation of the expression of phosphorylated Akt, phosphorylated GSK-3β, and nuclear β-catenin was inhibited by the PI3K/Akt blocking drug, LY294002. Combined treatment with A740003 and LY294002 did not result in additional inhibitory effects in comparison to those observed in the presence of LY294002 alone (Fig. 4a). The qRT-PCR and Western blot analysis results showed that EMF exposure increased the expression of osteogenic markers (RUNX2, ALP, and OPN) at both the mRNA and protein levels. The EMF-dependent increase in osteogenic marker expression was partially inhibited by A740003 and LY294002, although the inhibitory effects of LY294002 were superior. The inhibition of osteogenic marker expression-mediated A740003+LY294002 was comparable with that medicated by LY294002 alone (Fig. 4b, c). Alizarin red S staining of h-MSCs after osteogenic induction for 7 days revealed a greater number of calcium nodules in the EMF group than were observed in the other treatment and control groups. The numbers of calcium nodules in the EMF+A740003, EMF+LY294002, and EMF+A740003+LY294002 groups were greater than those in the control and A740003 groups but less than the number in the EMF group (Fig. 4d). These results suggested that P2X7 acts upstream of Akt/GSK-3β/β-catenin signaling and is partially responsible for EMF-induced osteogenic differentiation.

### P2X7 activation enhanced the pro-osteogenic effect of EMFs

**Fig. 5:**
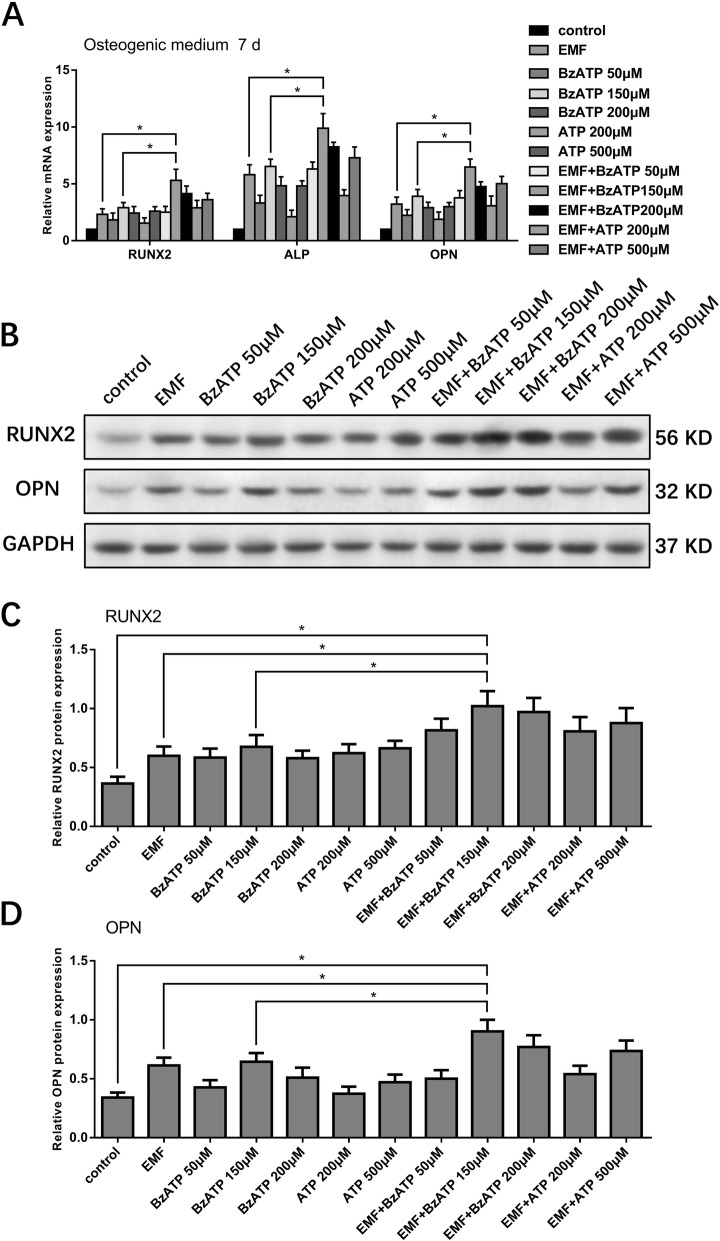
Effect of EMF exposure combined with P2X7 agonists on osteogenic markers. **a** qRT-PCR and **b**–**d** Western blot analysis of the effect of EMF exposure and/or BzATP (50, 150, and 200 μM) and ATP (200 and 500 μM) on osteogenic induction of h-MSCs. Data represent means ± SD. Statistically significant differences are indicated; *n* = 3; *P < 0.05, vs control

Having confirmed that P2X7 participated in the pro-osteogenic effect of EMF, we next investigated the ability of P2X7 activation to enhance the pro-osteogenic effect of EMF using the synthetic P2X7 agonist BzATP or its natural ligand ATP. After osteogenic induction of osteogenic differentiation of h-MSCs for 7 days, EMF exposure in the presence of either of the P2X7 activators upregulated the expression of osteogenic markers (RUNX2, ALP, and OPN) at both the mRNA and protein levels compared with the effects of EMF stimulation alone. Among all the groups, EMF combined with 150 μM BzATP exhibited the optimal upregulation of osteogenic marker expression, with levels of expression that were significantly higher than those observed following treatment with EMF or 150 μM BzATP alone (Fig. 5a–d). These results suggested that the pro-osteogenic effect of EMF on h-MSCs is additively enhanced by P2X7 activation.

### EMFs combined with P2X7 activation effectively protected the trabecular micro-architecture in OVX rats

**Fig. 6:**
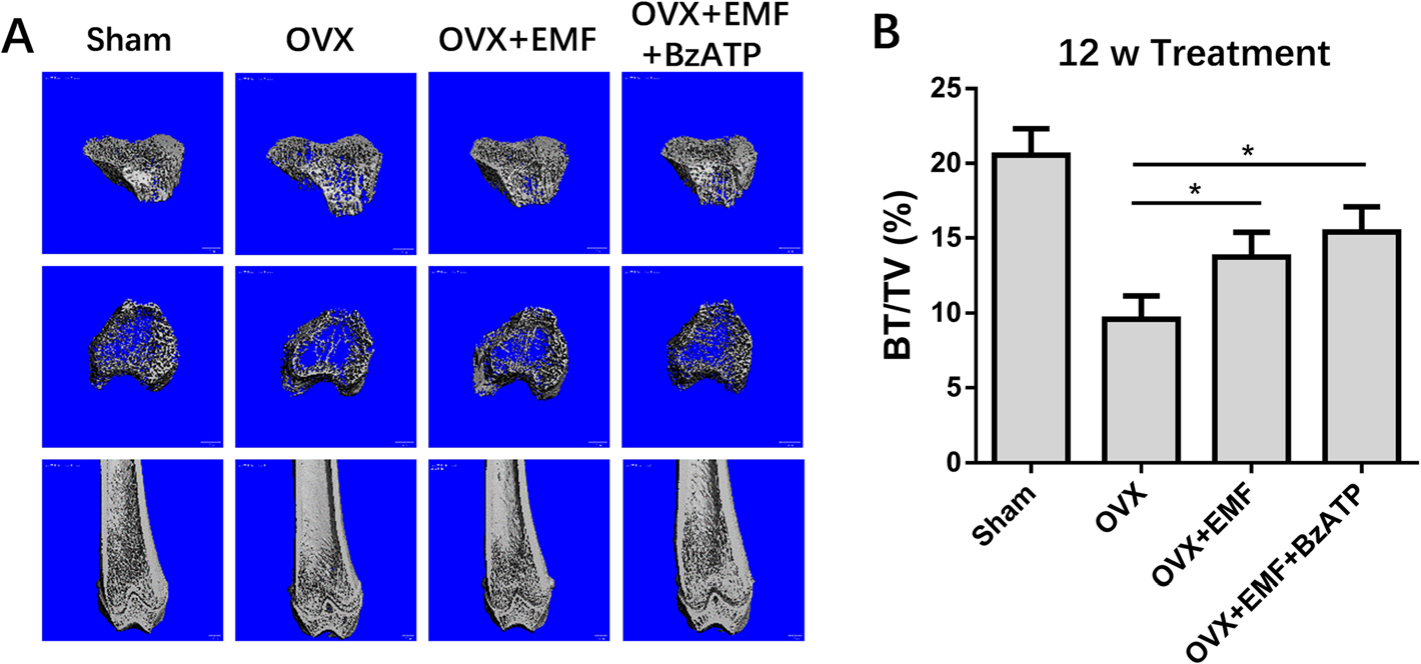
Effects of 12 weeks of EMF exposure on trabecular bone micro-architecture in the distal femora and proximal tibiae. **a** Trabecular bone micro-architecture from 2.0 mm height in the proximal tibiae and coronal observation in the distal femora in mice the sham-operated, OVX, OVX+EMF, and OVX+EMF+BzATP groups. **b** Micro-CT analysis quantification of BV/TV (bone volume/tissue volume) in the proximal region of the tibiae. Data represent means ± SD. Statistically significant differences are indicated; *n* = 6; *P < 0.05, vs control

The pro-osteogenic effect of EMFs medicated by increasing P2X7 expression was investigated in vivo using a rat model of rat osteoporosis induced by ovariectomy (OVX). OVX model rats were randomly allocated to four groups: sham-operated controls, OVX, EMF (15 Hz/1 mT, 4 h/day), and EMF+BzATP (0.25 mg/kg). After 12-week treatment, the trabecular micro-structure was evaluated by micro-CT imaging and the bone volume/total volume (BV/TV) was calculated to reflect trabecular bone volumes. Compared to sham-operated rats, OVX rats displayed a 50% decrease in trabecular bone volume. EMF treatment increased the trabecular bone volume of OVX rats by 30% and was further increased to 50% by EMF exposure combined with BzATP administration (Fig. 6a, b). These results suggested that P2X7 activation additively augmented the therapeutic effect of EMF on osteoporosis in vivo.

## Discussion

Osteoporosis is a systemic disease, characterized by loss of bone mineral density (BMD) and disruption of bone micro-architecture, leading to increased bone fragility which is a common and costly condition among postmenopausal women [1]. EMF treatment, considering its safety, efficacy, and noninvasion, has been studied and applied in orthopedics for many years and is commonly used in the clinical to promote fracture healing. In the last few years, EMFs have been reported to positively affect the balance of osteoblast and adipocyte differentiation of mesenchymal stem cells [13] and the balance between bone resorption and bone formation [16], which play important roles in the pathogenesis of osteoporosis. However, the parameters of EMF are difficult to standardize and the underlying mechanisms of their effects remain to be fully elucidated, which will limit the further use and improvement of EMF-associated anti-osteoporosis treatments.

Among the possible molecular mechanisms of EMF treatments, one of the popular theories is that the therapeutic effect of EMFs against osteoporosis is mediated by the production of excessive voltage-gated calcium channel (VGCC) activity, leading to increased intracellular Ca^2+^ and activation of the Ca^2+^/NO/cGMP/PKG pro-osteogenic signaling pathway [32]. Similarly, as a ligand-gated Ca^2+^-permeable cation channel, the purinergic receptor P2X7 is also implicated in skeletal remodeling and mechano-transduction [24]. In different species, P2X7 is expressed by both osteoblasts [33] and osteoclasts [34, 35] and mediates interactions between osteoblast and osteoclast through calcium oscillation [36] and other signalings [37]. One of the major effects of P2X7 activation in osteoblasts is to promote osteoblast proliferation and calcium deposition [26], which is involved in a range of different signaling pathways including PI3K [38], c-fos [39], ERK [40], and COX [26].

Based on the similarities between the biological effects of EMF and P2X7, we hypothesized that there is a correlation or interaction between EMF and P2X7. Thus, we compared the expression of P2X7 in h-MSCs with or without EMF stimulation. No changes in P2X7 mRNA or protein levels were detected following EMF exposure of h-MSCs cultured in DMEM/F12 for 21 days, indicating that EMF exposure does not affect P2X7 expression under regular culture conditions. Previous studies showed a steady, but low-level increase in P2X7 expression in rat BMSCs cultured in basic medium, whereas expression was increased fivefold after osteogenic induction for 7 days [41]. We speculated that the P2X7 gene transcription is not active under regular culture conditions, leading to an absence of susceptibility to EMFs, whereas prior to osteogenic differentiation of MSCs, during which the P2X7 gene is actively transcribed. Hence, we examined P2X7 expression in h-MSCs during osteogenic differentiation with or without EMF exposure. To our surprise, we found that the mRNA and protein content of P2X7 in the osteogenic medium+EMF group was significantly higher than that in the group treated with osteogenic medium alone at every time point from 3 to 21 days. These observations indicate that the upregulation of P2X7 expression during osteogenic differentiation of MSCs can be further enhanced by EMF exposure.

It has been reported that exposure to 15 Hz/1 mT EMFs promotes the osteogenic differentiation of MSCs and produces therapeutic effect on osteoporosis, although the underlying mechanism is unclear. In this study, we confirmed the pro-osteogenic effect of 15 Hz/1 mT EMFs in h-MSCs. To investigate the potential role of EMF-induced upregulation of P2X7 expression in this anti-osteoporosis effect, we analyzed the effects of EMF stimulation combined with P2X7 blockade on h-MSCs. The P2X7 antagonist did not obviously suppress the basal osteogenic differentiation of h-MSCs, but significantly lowered the pro-osteogenic effect of EMFs. These data indicate that the EMF-stimulated overexpression of P2X7 enhances the osteogenic capacity of h-MSCs and is partially responsible for the EMF-increased osteogenic differentiation. Thus, our results confirmed the pro-osteogenic effect of 15 Hz/1 mT EMFs in h-MSCs; this raised the question of which signaling pathways are involved in the mechanism by which the EMF-increased P2X7 promotes osteogenic differentiation of h-MSCs.

MSC differentiation, unlike most other biological processes, is manifested as dramatic cell type changes at epigenetic, transcription, and translation levels. Therefore, it is involved in numerous biological signalings, cytokines, and transcription factors. A number of signaling pathways have been identified to be related to MSC differentiation including wingless-type MMTV integration site (Wnt) signaling, transforming growth factor-beta (TGF-β)/bone morphogenic protein (BMP), MAPKs, Hedgehogs (Hh), Notch, and RhoA/ROCK pathways [42–44]. Among these signaling pathways, Wnt signaling plays an important role to regulate osteogenic differentiation of MSCs. In the canonical Wnt signaling, β-catenin is the key transcriptional coactivator which transmits extracellular signals to the nucleus and activates target genes [45].

Another important signaling pathway, the PI3K/Akt axis, is also a key signal to regulate cell survival, proliferation, and differentiation, which interacts with other signaling pathways and transcriptional networks controlling MSC differentiation [46].

GSK3β, a common downstream partner, is the intersection of the PI3K/Akt and the Wnt/β-catenin signaling pathways. Akt negatively regulates GSK-3β kinase activity by direct phosphorylation at Ser9 [47, 48]. When GSK-3β is active, the Wnt signaling pathway is inactivated and β-catenin is constitutively phosphorylated by a destruction complex composed of Axin, APC, and GSK-3β, leading to a low cellular level of β-catenin. When GSK-3β is inactivated by Akt, the β-catenin accumulates in the cytosol and translocates to the nucleus to activate downstream target genes such as RUNX2 [49, 50].

In the present study, EMF treatment activated the Akt/GSK3β/β-catenin signaling pathway, increased the expression of osteogenesis-related markers, and promoted osteogenic differentiation of h-MSCs. These effects were partially suppressed by either the P2X7 antagonist A740003 or the PI3K/Akt inhibitor LY294002, although the inhibitory effect of LY274002 was superior to that of A740003. Compared to the effects of administration of the individual drugs, combined treatment with A740003 and LY294002 did not cause an additional reduction in the EMF-induced effects, suggesting that EMF-induced P2X7 overexpression functions as an upstream regulator of the Akt/GSK3β/β-catenin pathway to promote osteogenic differentiation of h-MSCs.

Based on our findings, we optimized the therapeutic effect of EMFs by auxiliary activation of P2X7. Our in vitro data confirmed that application of P2X7 agonists (ATP or BzATP) can additively enhance the osteogenic effect of EMFs. Furthermore, we successfully translated this in vitro phenomenon into in vivo therapies, in which EMFs combined with BzATP administration produced a stronger therapeutic effect against osteoporosis in comparison with that achieved by EMF exposure alone.

## Conclusion

**Fig. 7:**
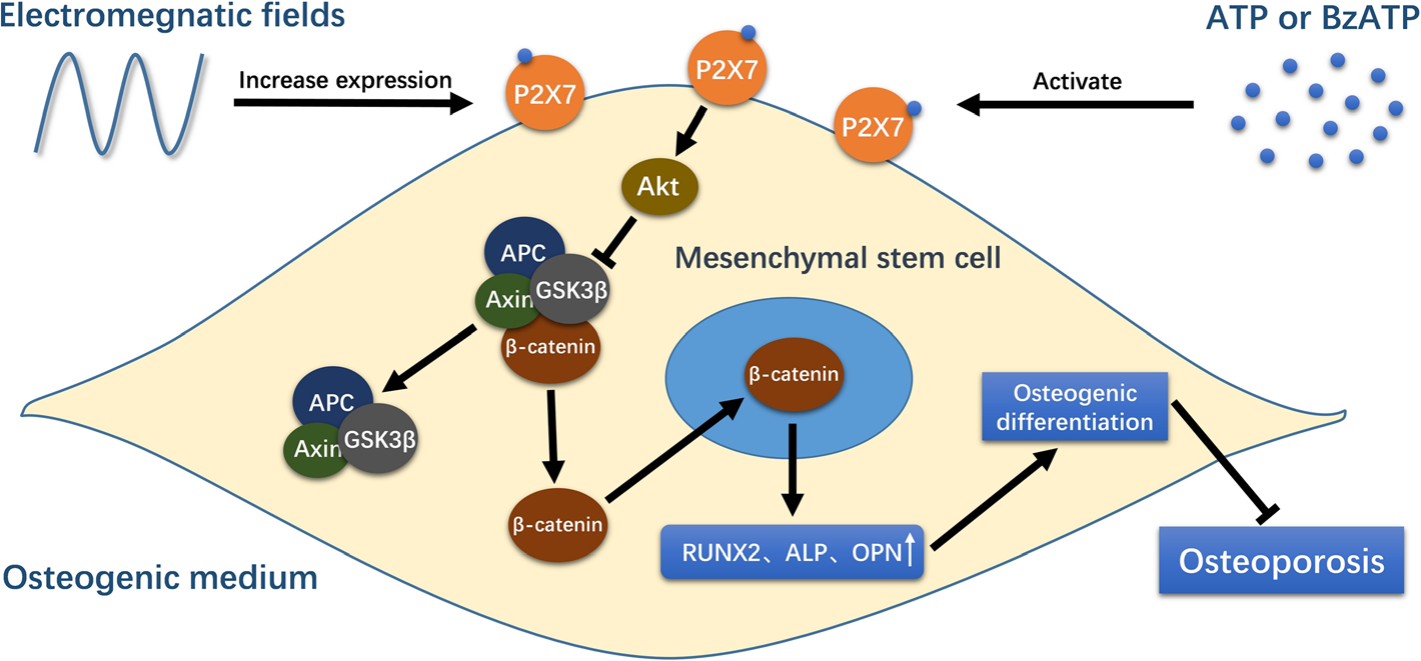
Diagrammatic depiction of EMF-induced upregulation of P2X7 expression and the downstream pro-osteogenesis effect. P2X7 expression is upregulated in h-MSCs exposed to EMFs during osteogenic differentiation. The EMF-induced upregulation of P2X7 can be activated by P2X7 agonists, ATP or BzATP. P2X7 ligation activates the Akt/GSK3β/β-catenin axis to induce osteogenic differentiation of MSCs (accompanied by upregulated expression of osteogenic markers) and inhibit osteoporosis

In conclusion, the evidence obtained in this study indicates that P2X7 expression is upregulated in h-MSCs exposed to EMFs during osteogenic differentiation. The pro-osteogenic and anti-osteoporotic effects of EMFs partially benefit from increasing P2X7 expression. The EMF-induced P2X7 overexpression promotes h-MSC osteogenic differentiation via the Akt/GSK3β/β-catenin signaling pathway. We also demonstrate that P2X7 agonist treatment may be an effective approach to additively augment the therapeutic effect of EMFs (Fig. 7).

## Supplementary information

**Additional file 1.** EMF device, identification of human bone marrow mesenchymal stem cells, and primer sequences for quantitative RT-PCR.

## Data Availability

The datasets used and/or analyzed during the current study are available from the corresponding author on reasonable request.

## Abbreviations

EMFs: Electromagnetic fields
P2X7: Purinergic receptor P2X7
h-MSCs: Human bone marrow mesenchymal stem cells
OVX: Ovariectomy
BV/TV: Bone volume/total volume
