# Supplementary material for: Electromagnetic field treatment increases purinergic receptor P2X7 expression and activates its downstream Akt/GSK3β/β-catenin axis in mesenchymal stem cells under osteogenic induction
Source: Stem Cell Res Ther. 2019 Dec 21;10:407. doi: 10.1186/s13287-019-1497-1 (PMC6925409; doi:10.1186/s13287-019-1497-1)
Supplement: Supplementary file 1 — Additional file 1. EMF device, identification of human bone marrow mesenchymal stem cells, and primer sequences for quantitative RT-PCR. [file 13287_2019_1497_MOESM1_ESM.docx]

**EMF Device**

The EMF producing device was designed and manufactured by the Naval University of Engineering of China (Wuhan, China). It comprised a waveform generator, amplifier, oscilloscope, and Helmholtz coils. The waveform generator created the signals, which, after being amplified, were output to the coils. The Helmholtz coils producing EMF were wound with 0.8mm diameter, coated copper wire. The coils were 30 cm in diameter, 15 cm apart. For the in vitro experiments, the Helmholtz coils were placed perpendicular to the horizontal plane in a CO_2_ incubator (Thermo Scientific, Wilmington, DE; 5% CO_2_, 37 °C and 100% humidity). For the in vivo experiments, the Helmholtz coils were placed in 26 ℃ and ventilation environment and the rats were contained in plexiglas cages (length ¼ 35 cm, width ¼ 30 cm, height ¼ 45 cm) individually and allowed ad libitum access to clean tap water and standard rodent chow (Animal Center of Tongji Medical College) during the whole period of experiment. The device could produce a magnetic flux density range of 0.0-5.0mT and a frequency range of 0-100 Hz. sinusoidal electromagnetic field was introduced as it showed a satisfying effect in our previous studies（Stem cell research & therapy. 2018;9:143; Bioelectromagnetics. 2017;38:137-150）. Magnetic field amplitude of the activated coils was measured using a gauss meter (GM55A; TinDun Industry, Shanghai, China). The uniformity of EMF was approximately 90% in the 7 cm of the spherical region (from the coil center to the origin).


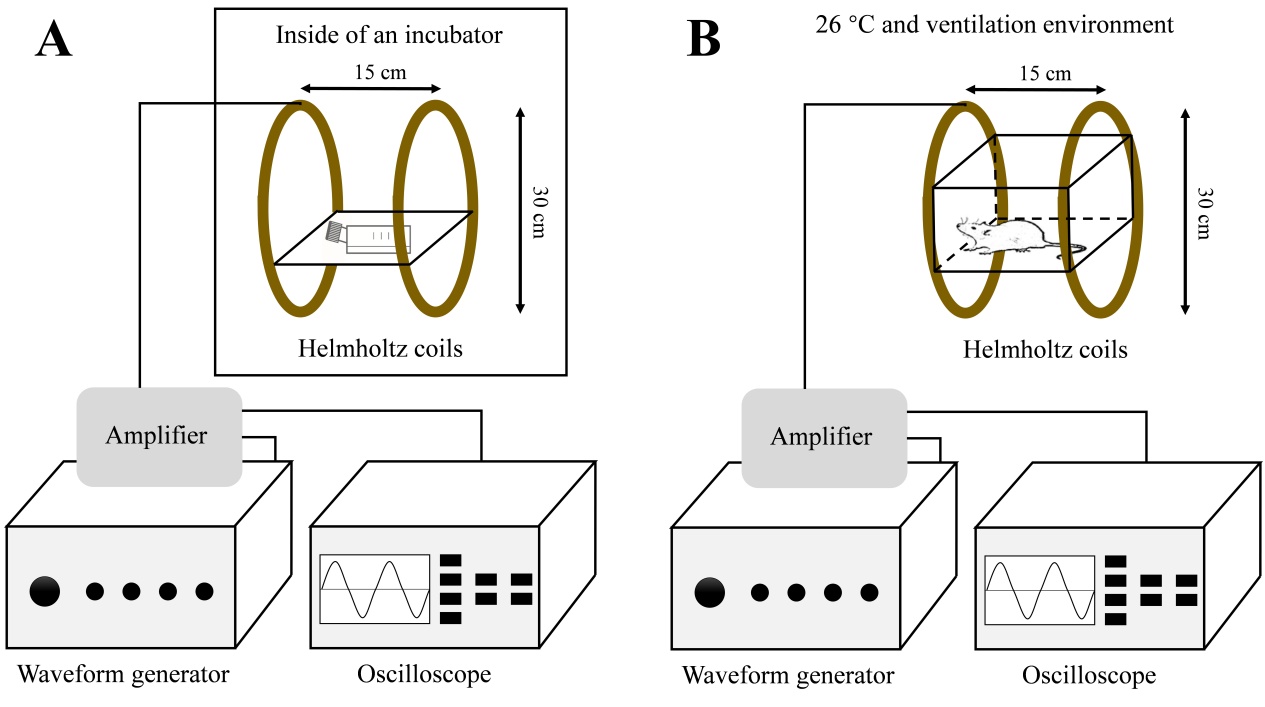


Figure S1. Representation of the device used to generate the electromagnetic fields (EMF). The device consists of four main parts: a waveform generator, amplifier, oscilloscope, and Helmholtz coils. (A)For the *in vitro* experiments, the Helmholtz coils were placed in a 5% CO2 and 37 °C incubator. Cells were placed in the center of the coils where the EMF is uniform (90%) and the temperature is nearly the same as other incubators (within 0.2–0.8 °C). (B)For the in vivo experiments, the Helmholtz coils were placed in to 26 °C and ventilation environment and the rats were contained in plexiglas cages.

**Identification of human bone marrow mesenchymal stem cells**

Human bone marrow MSCs were purchased from Cell Bank of Chinese Academy of Sciences (Shanghai, China). The cells were identified by detecting cell surface markers and the MSC multipotent potential for differentiation toward the adipogenic, osteogenic, and chondrogenic lineages.

Flow cytometry was performed to determine the surface markers expression on undifferentiated MSCs. We used CD105, CD73, CD90, CD45, CD34 and HLA-DR antibodies (Abcam, Cambridge, UK). The undifferentiated MSCs were positive for CD105, CD73 and CD90. CD45, CD34 and HLA-DR were not expressed in MSCs (Supplementary Fig. 2A).

To examine the multilineage capacity of MSC, cells were differentiated toward the adipogenic, osteogenic, and chondrogenic lineages using lineagespecific induction factors. To determine if MSCs undergo adipogenesis, cells were cultured in adipogenic medium (Cyagen Biosciences, Wuhan, China) and stained with Oil Red-O. MSCs cultured in adipogenic medium were induced toward the adipogenic lineage as early as 2 weeks’ post-induction. A significant fraction of the cells contained multiple, intracellular lipid-filled droplets that accumulated Oil Red-O (Supplementary Fig. 2B). Differentiation of MSC into osteoblasts was induced by osteogenic medium (Cyagen Biosciences, Wuhan, China). To confirm osteogenic differentiation, calcification of the extracellular matrix (ECM) was assessed in MSC using Alizarin red stain. Calcification appears as red regions within the cell monolayer. Consistent with osteogenesis, several red regions, indicative of a calcified ECM, were observed in MSCs treated for 2 weeks in osteogenic medium (Supplementary Fig. 2B). Chondrogenic differentiation was induced using chondrogenic medium (Cyagen Biosciences, Wuhan, China). To verify the chondrogenic capacity, MSCs were cultured in chondrogenic medium for stained with Alcian Blue which use to access proteoglycans accumulation. Blue regions appeared in MSCs which cultured 2 weeks in chondrogenic medium (Supplementary Fig. 2B).

The above results suggested that the human bone marrow MSCs in our study conformed the surface markers expression of mesenchymal stem cells and had the multilineage capacity, which confirmed the authenticity and reliability of the cells we used.


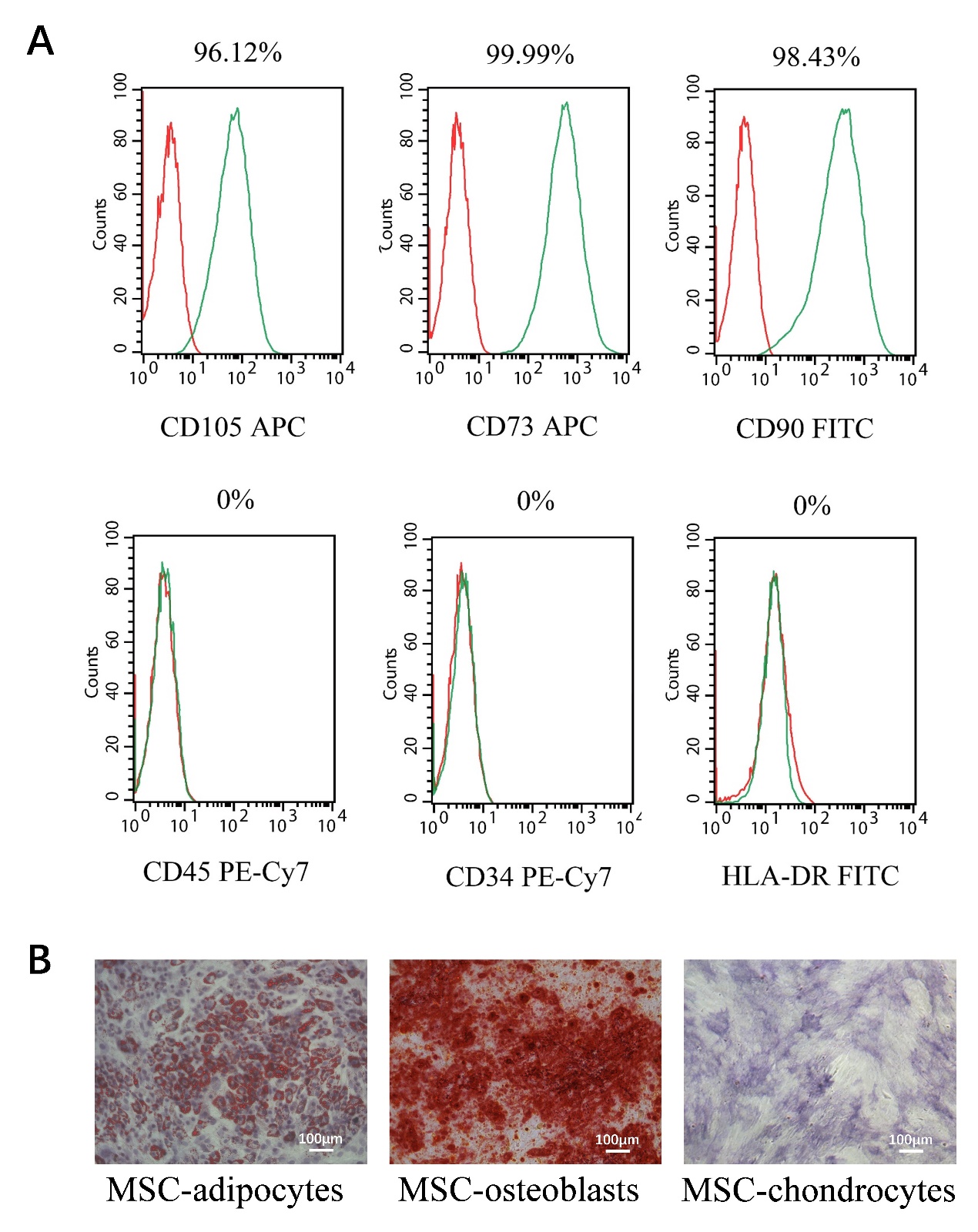


Figure S2. Identification of human bone marrow mesenchymal stem cells (MSCs) by detecting cell surface markers and the MSC multipotent potential for differentiation toward the adipogenic, osteogenic, and chondrogenic lineages. (A) Expression of mesenchymal stem cell marker in MSCs was determined by Flowcytometry. (B) MSCs were cultured for 2 weeks in adipogenic medium, osteogenic medium and chondogenic medium and stained with Oil Red O, Arizarin S and Alcian Blue to identify differentiation.

**Primer sequences for quantitative RT-PCR**

Supplementary Table 1 Primer sequences for quantitative RT-PCR

| Gene | Forward (5’–3’) | Reverse (5’–3’) |
| --- | --- | --- |
| GAPDH | GGAGCGAGATCCCTCCAAAAT | GGCTGTTGTCATACTTCTCATGG |
| P2X7 | GTCTGGCCGCTGTGTTCATCG | CTGTATCTCCTCTGGT TGTCC |
| RNUX2 | TCAACGATCTGAGATTTGTGGG | GGGGAGGATTTGTGAAGACGG |
| ALP | ACTGGTACTCAGACAACGAGAT | ACGTCAATGTCCCTGATGTTATG |
| OPN | GAAGTTTCGCAGACCTGACAT | GTATGCACCATTCAACTCCTCG |
